# Task-Specific Vocal Acoustic Markers Reveal Amyloid Burden

**DOI:** 10.1093/geroni/igaf122.4206

**Published:** 2025-12-31

**Authors:** Hyunsun Ham, Jiwon Son, Keun You Kim, So Yeon Jeon, Jun-Young Lee

**Affiliations:** Seoul National University, Republic of Korea; Seoul National University, Republic of Korea; Yonsei University College of Medicine, Seoul, Republic of Korea; Seoul Metropolitan Government-Seoul National University (SMG-SNU) Boramae Medical Center, Seoul, Republic of Korea; Seoul Metropolitan Government-Seoul National University (SMG-SNU) Boramae Medical Center, Seoul, Republic of Korea

## Abstract

This study investigates whether task-specific vocal acoustic features can reliably predict amyloid PET positivity in Alzheimer’s disease. While prior works often relied on a single task such as picture description, we systematically designed five distinct speech tasks, each designed at a different linguistic level—from acoustic-phonetic to discourse-level—to examine how the underlying pathology manifests in speech production at varying linguistic levels. Seventy-five participants completed five speech tasks, including 46 amyloid-negative and 29 amyloid-positive individuals, as determined by PET imaging. From each task, we extracted 162 hand-crafted acoustic features using an optimized clinical speech analysis pipeline. These features included spectral features(MFCCs), prosodic features (pitch, intensity, speech rate), and voice quality measures (jitter, shimmer, HNR). Recursive feature elimination and XGBoost were employed for feature selection, followed by task-specific classification using multiple binary classifiers. Among the tasks, the Sequential Motion Rate task yielded the highest accuracy(79%). Pitch-related features consistently ranked among the most important across all tasks, suggesting their robustness regardless of linguistic level. Prosodic features demonstrated increased discriminative power in higher-order tasks, whereas voice quality features were particularly salient in tasks emphasizing phonation and articulation. These findings highlight that vocal acoustic markers from specific speech tasks can effectively reveal amyloid burden, with distinct acoustic patterns depending on the linguistic complexity of the task. These task-dependent speech markers may reflect early neurodegenerative changes, supporting a link between amyloid pathology and speech production.

